# Different approaches to investigate sleep apnea

**DOI:** 10.5935/1984-0063.20180012

**Published:** 2018

**Authors:** Monica L. Andersen

**Affiliations:** 1 Departmento de Psicobiologia, Universidade Federal de São Paulo, Brazil.

Sleep disorders, in particular sleep apnea, has been recognized as a public health problem. Obstructive sleep apnea (OSA) is a common condition in subjects from both sex and all ages^1,2^, and it is associated with higher morbidity and mortality^3^^,^^4^. In Brazilian population, the presence of severe OSA (apnea-hypopnea index - AHI≥15) was predictor of hospitalizations and/or demand for emergency services^5^. Recently, Scalzitti et al.^5^ analyzed more than 22,000 patients to identify factors that contribute to hospital readmission within 30 days of discharge. Authors found that OSA was an independent risk factor for readmission^5^.

OSA consequences to subjects’ health and well-being warranted further investigation of the diverse factors that could interfere with OSA treatment and prognostic. Moreover, the clinical, social and economic burden of sleep disorders indicate the importance to explore OSA with a multidisciplinary perspective and using a broad approach. In this number of Sleep Science, four articles looked into OSA using different approaches, aiming to analyze the associated factors with the disorder risk and severity, and the respective treatments outcomes.

We highlight the studies that explored OSA diagnostic and treatment using alternative means. This approach could be helpful in clinical situations with limited/no access to the gold-standartd tools. Kale et al.^6^ investigated the association of oral characteristics, as maxillary arch constriction, facial profile and tongue size, with the risk of OSA. Authors applied the STOP-BANG questionnaire in a sample of patients admitted in a specialized hospital for dental treatment. Results demonstrated that the risk of being classified as high risk for OSA was more than 2 times higher in participants that had neck circumference >40cm, Mallampati score class 3 or 4, large tongue, and deep palatal vault. These results are very interesting due to the clinical applicability. The oral parameters analyzed in the study could be used in dental clinical practice as an initial screening of OSA risk, enable to reduce the time for polysomnography exam, diagnosis and treatment. The use of oral and orthodontic evaluations is also applied to OSA treatment.

In a Case Report, Guimaraes et al.^7^ described a significant improvement in AHI after mandibular advancement device treatment in a CPAP-intolerant male patient. After two years of treatment, patient showed a reduction of the AHI from 80.5 events/h to 8 events/h, in addition to improvements in oxygen saturation parameters and arousal index. The article demonstrates the importance to use alternative therapies to OSA patients with no CPAP adherence.

OSA is highly prevalent in society and strongly associated to several comorbidities. Increase the access and the response to OSA treatment, and facilitate the disease diagnostic are challenges in Sleep Medicine. Studies in this field are essential to the discussion and development of new clinical and research strategies.
